# Objectively recorded physical activity in pregnancy and postpartum in a multi-ethnic cohort: association with access to recreational areas in the neighbourhood

**DOI:** 10.1186/s12966-016-0401-y

**Published:** 2016-07-07

**Authors:** Kåre Rønn Richardsen, Ibrahimu Mdala, Sveinung Berntsen, Yngvar Ommundsen, Egil Wilhelm Martinsen, Line Sletner, Anne Karen Jenum

**Affiliations:** Norwegian National Advisory Unit on Women’s Health, Oslo University Hospital, Oslo, Norway; Faculty of Health Sciences, Oslo and Akershus University College of Applied Sciences, PB 4 St Olavs Plass, N-0130 Oslo, Norway; Faculty of Medicine, Department of General Practice, Institute of Health and Society, University of Oslo, Oslo, Norway; Faculty of Health and Sport Sciences, University of Agder, Kristiansand, Norway; Department of Coaching and Psychology, Norwegian School of Sport Sciences, Oslo, Norway; Clinic Mental Health and Addiction, Oslo University Hospital, Oslo, Norway; Institute of Clinical Medicine, University of Oslo, Oslo, Norway; Department of Pediatric and Adolescents Medicine, Akershus University Hospital, Lørenskog, Norway

**Keywords:** Physical activity, Urban planning, Neighbourhoods, Pregnancy, Geographic information systems, Ethnic groups

## Abstract

**Background:**

Physical activity may reduce the risk of adverse pregnancy outcomes; however, compared to non-pregnant women, a lower proportion of pregnant women meet the physical activity guidelines. Our objectives were to explore overall changes and ethnic differences in objectively recorded moderate-to-vigorous intensity physical activity (MVPA) during pregnancy and postpartum and to investigate the associations with objective and perceived access to recreational areas.

**Methods:**

We analysed 1,467 person-observations from 709 women in a multi-ethnic population-based cohort, with MVPA data recorded with the SenseWear™ Pro^3^ Armband in early pregnancy (mean gestational week (GW) 15), mid-pregnancy (mean GW 28) and postpartum (mean postpartum week 14). MVPA was limited to bouts ≥10 min. Women were nested within 56 neighbourhoods defined by postal code area. We derived neighbourhood-level objective access to recreational areas (good vs limited) by geographic information systems. We collected information about perceived access (high vs low perception) to recreational areas in early pregnancy. We treated ethnicity, objective and perceived access as explanatory variables in separate models based on linear mixed effects regression analyses.

**Results:**

Overall, MVPA dropped between early and mid-pregnancy, followed by an increase postpartum. Western women performed more MVPA than women in other ethnic groups across time points, but the differences increased postpartum. Women residing in neighbourhoods with good objective access to recreational areas accumulated on average nine additional MVPA minutes/day (*p* < 0.01) compared with women in neighbourhoods with limited access. Women with perceptions of high access to recreational areas accumulated on average five additional MVPA minutes/day (*p* < 0.01) compared with women with perceptions of low access. After mutual adjustments, perceived and objective access to recreational areas remained significantly associated with MVPA. The association between MVPA and access to recreational areas did not differ by time point, ethnic group or socio-economic position.

**Conclusions:**

In all ethnic groups, we observed a decline in MVPA between early and mid-pregnancy. However, at both time points during pregnancy, and especially three months postpartum, Western women were more physically active than ethnic minority women. In all ethnic groups, and at all three time points, both objective and perceived access to recreational areas were positively associated with MVPA levels.

**Electronic supplementary material:**

The online version of this article (doi:10.1186/s12966-016-0401-y) contains supplementary material, which is available to authorized users.

## Background

There is growing evidence that physical activity (PA) during pregnancy may reduce the risk of gestational diabetes, excessive gestational weight gain and maternal depressive symptoms [1]. According to PA guidelines for the general adult population, 150 MVPA minutes/week performed in bouts ≥10 minutes is recommended [2]. PA guidelines for pregnant women have also adopted this recommendation [3]. Nevertheless, the proportion who achieve the recommended 150 MVPA minutes/week is lower among pregnant than non-pregnant women, and PA levels decline as pregnancy progresses [4]. We have previously shown that MVPA levels in early pregnancy are particularly low among women of South Asian and Middle Eastern origin [5], and lower MVPA levels among ethnic minority women have also been reported elsewhere [6]. Current knowledge about PA levels in pregnancy is predominantly based on self-reported data and hence estimates [7] as well as associations with potential explanatory variables are prone to bias.

In contrast with health behaviour models with a sole focus on individual characteristics, ecological models consider broader contexts [8]. The relationship between PA and the neighbourhood context is an emerging research field [9]. Recreational areas such as natural environments can influence PA via enhanced attitudes towards PA, perceived behavioural control and intention to engage in PA [10]. Neighbourhoods represent important arenas for recreational walking [11]. While participation in many types of PA drops during pregnancy, brisk walking becomes the most common type of MVPA [12]. It is thus reasonable to assume that good access to recreational areas may positively influence PA. Studies of non-pregnant women show that proximity to neighbourhood parks and green spaces is associated with PA [13, 14]. The majority of studies have employed individual perceptions as measures of access to recreational areas, but reliance on perceptions alone may induce bias and risk of reverse causality [15, 16].

We are unaware of studies of the association between perceived neighbourhood environment and PA in pregnancy or early postpartum, but qualitative studies have shown that heavy traffic and unsafe neighbourhood parks are perceived as PA barriers [17, 18]. Geographic information systems (GIS) can be used to incorporate relevant measures of distance and area size and provide objective measures of access to recreational areas [19], but to our knowledge, no study of PA in pregnancy has employed such GIS data. However, one study of PA during pregnancy used data on park access collected by observational methods [20].

While perceived and objective neighbourhood walkability may be dissimilar, they have independent effects on MVPA [21]. While perceptions of the environment may induce bias in studies of PA behaviour, proponents of ecological models argue that insight into PA behaviour depends on understanding the interplay among individual factors (e.g., ethnicity, family situation, socio-economic position), individuals’ perceptions of the environment (e.g., access, convenience) and objective characteristics of the environment (e.g., bike lanes, parks) [8].

In the present study, we first analysed MVPA changes and ethnic differences during pregnancy and early postpartum. Second, we examined associations between objective and perceived access to recreational areas. Third, we examined MVPA associations with objective and perceived access to recreational areas and potential effect modification of access by time point, ethnicity and socio-economic position. Finally, we examined the variation in MVPA that can be attributed to differences among neighbourhoods and among individuals.

## Methods

### Design and data collection

In the present study, we combined data at the individual-level with GIS-derived data on access to recreational areas at the neighbourhood-level. Individual-level data were from the Stork Groruddalen Cohort Study (Stork-G) of pregnant women living in three multi-ethnic city districts of Oslo. Participants were recruited between 2008 and 2010 at three child health clinics where they received antenatal care. Data collection was administered by trained midwives, and data were collected at three time points. In total, 74 % (*n* = 823) of the invited women were included and participated in the study in early pregnancy (mean gestational week (GW) 15), 772 participated in mid-pregnancy (mean GW 28), and 662 participated postpartum (mean postpartum week 14) [22]. At inclusion, the cohort was representative for women attending the child health clinics with respect to ethnicity and age [22]. Inclusion criteria were planned birth at either of two study hospitals, GW ≤20 and ability to communicate orally in Norwegian, Arabic, English, Sorani, Somali, Tamil, Turkish, Urdu or Vietnamese. Exclusion criteria were pre-gestational diabetes or other conditions necessitating intensive hospital follow-up during pregnancy. Collected data included questionnaire data collected during face-to-face interviews and objectively recorded PA data. Interviewing midwives had access to questionnaires in all the languages listed, and professional interpreters assisted during interviews if needed. All participants provided informed consent. The Regional Committee for Medical and Health Research Ethics for South Eastern Norway (ref: 2007/894) and the Norwegian Data Inspectorate approved the study protocol. The study methods are described in detail elsewhere [22]. GIS-derived data originated from postal code areas that overlapped with the residential areas served by the child health clinics. We linked individual-level data from Stork-G via participants’ postal codes to neighbourhood-level data. Data from the three time points were ineligible for analysis if a participant’s residency in early pregnancy was outside postal code areas with available GIS data or if the postal code was missing. We also excluded observations from mid-pregnancy and postpartum if the postal code was missing or different from early pregnancy, observations without valid PA data from any time point, and postpartum observations from women with pre-term birth (<GW 37) [Additional file 1].

### Objectively recorded physical activity

We collected PA data at all time points with the SenseWear™ Pro3 Armband (SWA) (BodyMedia Inc., Pittsburgh, Pennsylvania, USA) [23]. Women were asked to wear the SWA across the right triceps brachii over 4–7 days, and remove it only for water activities. We downloaded raw data integrated into 60-s epochs using the manufacturer’s software (SenseWear™ Professional Research Software Version 6.1, BodyMedia Inc.). MVPA minutes were extracted with SQL Server Management Studio (Microsoft®) and SQL Server Express version 11.0.5058.0 (Microsoft®) and limited to bouts ≥10 min at ≥3 metabolic equivalents (METs) (1 MET = 3.5 ml O_2_° kg^−1^∙min^−1^). A valid SWA day was defined as ≥19.2 h of SWA wear time. PA data from single time points were eligible if ≥2 valid SWA days were recorded [24].

### Objective access to recreational areas

We defined objective access to recreational areas according to Statistics Norway’s operationalization, expressed as the proportion of neighbourhood residents with residency <200 meters from a recreational area larger than 5,000 m^2^ and access along an eligible walking route (i.e., no need to cross roads with speed limits >30 km/h or metro-/rail tracks) [25]. Neighbourhoods were defined by the postal code areas recognized in 2008 by the national postal service (Posten Norge AS) to parallel the Stork-G data collection period. Analysts at Statistics Norway derived neighbourhood proportions of residents with access using ArcGis version 10.2.1 (ESRI, Redlands, CA, USA) based on geographical coordinates of home addresses for all residents within a postal code area, recreational-area access points and travel distance along eligible routes. We observed no linear dose–response association between access to recreational areas and MVPA. Hence, we explored different cut-off values and found that the 10^th^ percentile cut-point yielded the strongest bivariate association with MVPA and secured a minimum number of observations below the cut-point. In neighbourhoods below the 10^th^ percentile, the proportion of residents with access to recreational areas ranged from 0 to 41 % (“limited access”), while in neighbourhoods > 10^th^ percentile, the proportion ranged from 46 to 100 % (“good access”).

### Perceived access to recreational areas

Perceived access to recreational areas in early pregnancy was composed of four items originating from different previously used scales [26–28], with higher scores reflecting perceptions of better access. Item A measured on a six-point Likert scale measured perceived time to walk from home to recreational areas [27] but was reduced to four categories: 1 (don’t know or >30 min); 2 (11–20 or 21–30 min); 3 (6–10 min); and 4 (1–5 min). Items B-D used a four-point Likert scale ranging from 1 (totally disagree) to 4 (totally agree). Item B measured access to walking or bicycle paths; item C access to places/facilities appropriate for PA [28]; and item D access to safe and adequately lit locations for walking [26]. By principal components analysis, we extracted one component. Item A was removed due to weak loading on the extracted component. The reliability was moderate, reflecting the heterogeneous nature of the component (Cronbach’s α = 0.55) [29]. The component score showed a negatively skewed distribution, as two-thirds of the sample achieved the highest possible score. Hence, we used a mean-dichotomised outcome for the analysis (“perception of high access” versus “perception of low access”).

### Co-variates

We analysed participants’ age as a continuous measure. Seasonal categories were spring (March-May), summer (June-August), autumn (September-November) and winter (December-February). We defined ethnicity by the participant’s country of birth or her mother’s country of birth if her mother was born outside Europe or North America. Ethnic categories were Western (Western Europe and North America), South Asian, Middle Eastern and other ethnicity (for more details see Table 1). We classified parity into nulliparous and parous women. Using principal components analysis, we extracted one component with high reliability (Crohnbach’s α >0.7) reflecting socio-economic position (SEP) [30]. Higher SEP scores reflected higher socio-economic position. The SEP scores were normally distributed and treated as a continuous measure. The factors contributing most to the score were educational level, occupational class, employment status, renting tenure and rooms per person in the household.

**Table 1:** Baseline characteristics

	Valid postal code and valid PA data^1^ (*n* = 709)		Missing postal code and/or not valid PA data^1^ (*n* = 114)		
	Mean	SD	Mean	SD	*P*-value^2^
Age at inclusion (years)	30.1	4.8	28.5	4.9	<0.01
BMI pre-pregnancy	24.5	4.6	25.5	6.0	0.04
Socio-economic position (PCA-score)	0.04	1.0	−0.3	1.0	<0.01
	*n*	%	*n*	%	*P*-value^3^
Ethnicity					<0.01
Western^a^	308	(43.4)	28	(24.6)	
South Asian^b^	168	(23.7)	32	(28.1)	
Middle Eastern^c^	105	(14.8)	21	(18.4)	
Other ethnicity^d^	128	(18.1)	33	(28.9)	
Education					0.02
< 10 years	108	(15.3)	25	(22.5)	
10–12 years	274	(38.8)	50	(45.1)	
University or college	324	(45.9)	36	(32.4)	
Unknown	3		3		
Occupation					0.04
Elementary occupations and homemeakers	193	(27.8)	43	(39.1)	
Clerical/care occupations	245	(35.2)	37	(33.6)	
Manager/degree occupations	257	(37.0)	30	(27.3)	
Unknown	14		4		
Parity					0.15
None (nulliparous)	331	(46.7)	50	(43.9)	
1 (uniparous)	246	(34.7)	34	(29.8)	
≥ 2 (multiparous)	132	(18.6)	30	(26.3)	
Housing					0.53
Flat	566	(81.0)	91	(83.5)	
Semi-detached or detached housing	133	(19.0)	18	(16.5)	
Unknown	10		5		
Health pre-pregnancy					0.03
Poor/not too good	71	(10.1)	17	(15.6)	
Good	354	(50.4)	62	(56.9)	
Very good	278	(39.5)	30	(27.5)	
Unknown	6		5		
Smoking behaviour pre-pregnancy					0.51
Non-smoker	579	(82.1)	94	(84.7)	
Daily or irregular smoker	126	(17.9)	17	(15.3)	
Unknown	4		3		

^1^ Physical activity data based on ≥2 valid days from ≥1 time point

^2^ Independent samples t-test

^3^ Chi-square test

^a^ Valid data (*n* = 308): 287 from Norway, 13 from Denmark/Sweden, and remaining 9 from Western European countries and North America

^b^ Valid data (*n* = 168): 110 from Pakistan, 46 from Sri Lanka, 11 from India/Bangladesh

^c^ Valid data (*n* = 105): 33 from Iraq, 21 from Morocco, 20 from Turkey, 12 from Afghanistan, and remaining 19 from other countries in the region

^d^ Valid data (*n* = 128): 27 from Somalia, 12 from the Philippines, 14 from Vietnam, remaining 75 from 37 different countries

### Statistical analyses

Descriptive data are presented as means with standard deviation (SD), medians with interquartile range (IQR) and proportions. We analysed group differences between the analysed sample and participants ineligible for analysis in early pregnancy by t-tests and Chi-square tests. We assessed agreement between objective and perceived access to recreational areas by Kappa statistics.

#### Data structure

Level 1 consisted of repeated measurements (*n* = 1467) from time points 1–3. The repeated measurements were nested within women (*n* = 709) at level 2, and women were nested within neighbourhoods (*n* = 56 postal code areas) at level 3. Levels 2 and 3 were treated as random effects in the analyses.

#### Multilevel modelling

**Fig. 1:**
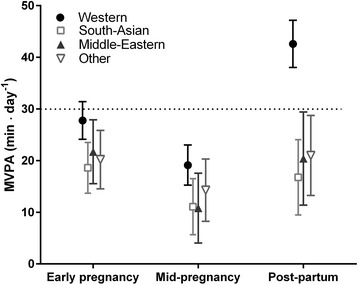
MVPA (minutes/day) for women in neighbourhoods according to ethnic group; Estimated marginal means with 95 % CI for women with Western, South-Asian, Middle Eastern and other ethnicity. Adjusted for season and week of PA monitoring, age and dependence between observations within individuals and within neighbourhoods. A significant interaction between ethnicity and time point manifests as a steeper increase in MVPA between mid-pregnancy and postpartum for Western women compared with the other ethnic groups

**Fig. 2:**
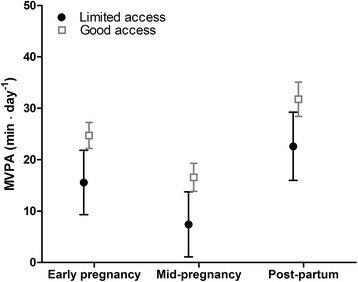
MVPA (minutes/day) for women in neighbourhoods with limited access vs good access to recreational areas; Estimated marginal effects with 95 % CI for limited and good objective access to recreational areas by time point. Adjusted for ethnicity, socio-economic position, season, parity, age, week of PA monitoring and dependence between observations within individuals and within neighbourhoods

**Fig. 3:**
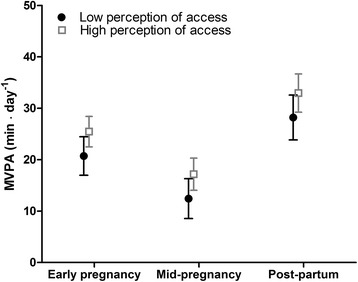
MVPA (minutes/day) for women with perceptions of high access vs low access to recreational areas; Estimated marginal effects with 95 % CI for high perceptions and low perceptions of access to recreational areas by time point. Adjusted for ethnicity, socio-economic position, season, parity, age, week of PA monitoring and dependence between observations within individuals and within neighbourhoods

We performed longitudinal analyses of MVPA using three-level linear mixed effects regression models to account for clustering of participants within neighbourhoods and clustering of repeated observations for individuals. For all models, we included a categorical variable indicating time point of PA recording (i.e. early pregnancy, mid-pregnancy or postpartum) and adjusted for gestational or postpartum week of PA recording (mean-centred at each time point) and season, which was a time-variant factor. Model specific co-variates are indicated in the description below:

First, we explored longitudinal changes in overall crude estimates of MVPA. Second, we estimated ethnic differences in longitudinal MVPA changes after adjustment for age. We included an interaction term between ethnicity and time point. We visualised the association between ethnicity and MVPA with a plot showing the marginal mean values with 95 % CI (Fig. 1). Third, we estimated the association between objective access to recreational areas and MVPA in pregnancy and postpartum by adjusting for ethnicity, SEP, parity and age and explored whether ethnicity, SEP and time point modified the association when adding interaction terms. Using the same procedure, we analysed the association between perceived access and MVPA. We visualised the adjusted associations between both measures of access to recreational areas and MVPA with plots showing the marginal effects with 95 % CI (Fig. 2 and 3). Finally, we explored the possible interaction between objective and perceived access to recreational areas.

We used estimates of intra-class correlation (ICC) obtained from a variance components model to describe the variation in MVPA that can be attributed to neighbourhoods and individuals. We also obtained adjusted ICC for models 1–3 (Table 2).

**Table 2:** Associations between access to neighbourhood recreational areas and moderate-to-vigorous intensity physical activity (minutes/day)

	Model 1^a^			Model 2^a^			Model 3^a^		
	β	*p*-value	95 % CI	β	*p*-value	95 % CI	β	*p*-value	95 % CI
Fixed effects									
Objective access to recreational areas (ref: limited access)									
Good access	9.14	<0.01	2.66, 15.62				9.06	<0.01	2.39, 15.74
Perceived access to recreational areas (ref: Low access)									
High access				4.75	0.02	0.68, 8.82	4.40	0.03	0.34, 8.45
Time point (ref: Early pregnancy)									
Mid-pregnancy	−8.15	<0.01	−10.94, −5.36	−8.28	<0.01	−11.16, −5.40	−8.23	<0.01	−11.11, −5.36
Post-partum	7.04	<0.01	3.65, 10.43	7.49	<0.01	4.01, 10.98	7.51	<0.01	4.03, 11.00
Random effects									
Intra-class correlation (%) neighbourhood-level	0.7			1.6			1.1		
Intra-class correlation (%) individual level	40.0			39.0			38.5		

^a^ Three-level linear mixed effects regression models with adjustment for week and season of time point for physical activity recording, socio-economic position, ethnicity, parity, age

#### Sensitivity analyses

We performed three types of sensitivity analyses: 1) we analysed sensitivity to number of SWA days using observations with ≥1 valid SWA day and ≥3 valid SWA days; 2) we assessed potential bias in complete case analyses by analysis of datasets generated by multilevel multiple imputation; and 3) we assessed robustness of the estimates to a possible missing not at random mechanism simulated by a worst-case scenario. For sensitivity analyses 2 and 3, see Additional file 2 for details. We performed multiple imputation in REALCOM-IMPUTE to account for cluster effects [31] and performed all other analyses in Stata 13 [32]. *P*-values ≤0.05 were considered statistically significant.

## Results

### Study sample

The study sample consisted of 709 participants with 1,467 observations from three time points: early pregnancy (*n* = 640), mid-pregnancy (*n* = 523) and postpartum (*n* = 304). The main reason for missing data was non-compliance with SWA instructions [see Additional file 1 for flow chart].

### Sample characteristics

Sample mean (SD) age was 30.1 (4.8) yrs, and mean (SD) body mass index (BMI) pre-pregnancy was 24.5 (4.6) kg/m^2^ (Table 1). The median (IQR) number of valid SWA days was 4 (3–4) in early- and mid-pregnancy and 3 (3–4) postpartum. Mean (SD) week of PA recording was GW 15.1 (3.2) in early pregnancy, GW 28.2 (1.4) in mid-pregnancy and week 13.7 (2.3) postpartum. Compared with participants without valid PA data and postal codes, the study sample was one year older and had lower BMI, with a higher proportion with Western ethnicity, higher education/social class and better subjective health (Table 1). MVPA median (IQR) values per time point for various characteristics are presented in Additional file 3.

### Association between objective and perceived access to recreational areas

Among residents in neighbourhoods with good objective access to recreational areas 68 % had perceptions of high access. Among residents in neighbourhoods with limited objective access to recreational areas 59 % had perceptions of high access. The Kappa value was 0.05 (95 % CI = −0.02–0.11), which indicates poor agreement.

### Ethnic differences in MVPA (age-adjusted)

Western women accumulated nine MVPA minutes/day more than South Asian women (*p* < 0.01) and eight MVPA minutes/day more than women with other ethnicity (*p* = 0.02) during early pregnancy (Fig. 1). Both South Asian (*p* = 0.02) and Middle Eastern (*p* = 0.03) women accumulated eight MVPA minutes/day less than Western women in mid-pregnancy. There was a significant interaction (<0.01) between ethnicity and time point, with a steeper increase in MVPA between mid-pregnancy and postpartum among Western women relative to the other ethnic groups (Fig. 1). Postpartum, Western women accumulated 26 MVPA minutes/day more than South Asian women (*p* < 0.01) and 22 MVPA minutes/day more than Middle Eastern women (*p* < 0.01).

### Associations between access to recreational areas and MVPA (adjusted)

On average, participants residing in neighbourhoods with limited objective access to recreational areas accumulated nine MVPA minutes/day less compared to participants in neighbourhoods with good objective access (*p* < 0.01) (Model 1, Table 2 and Fig. 2). Participants with perceptions of low access to recreational areas accumulated on average five MVPA minutes/day less compared to participants with perceptions of high access (*p* = 0.02) (Model 2, Table 2 and Fig. 3). The associations between MVPA and perceived and objective access were not modified by time point, ethnicity or SEP (not shown). After mutual adjustment for objective and perceived access to recreational areas, the associations between MVPA and objective (*p* < 0.01) and perceived access (*p* = 0.03) remained significant (Model 3, Table 2). The estimate for objective access remained unchanged while it dropped by approximately 10 % for perceived access in this model (Model 3, Table 2). We observed no interaction between objective and perceived access to recreational areas. A table showing estimates for models 1–3 with co-variates is provided in Additional file 4.

The ICC in the empty model (without explanatory variables) showed that 39 % of the total variation in MVPA can be attributed to differences among individuals and 2.2 % to differences among neighbourhoods. In the adjusted models the MVPA variation attributed to differences among neighbourhoods was 0.7 % in model 1, 1.6 % in model 2 and 1.1 % in model 3 (Table 2).

### Sensitivity analyses

Tests of sensitivity to the number of SWA days using ≥1 and ≥3 valid SWA days yielded similar results as original models 1–3. Analyses of imputed datasets replicated the results showing ethnic differences in MVPA in pregnancy and postpartum. The interaction between ethnicity and time point was no longer significant, but the confidence intervals indicated a trend [Additional file 2, Table 4a]. The associations between MVPA and objective and perceived access to recreational areas were supported by analysis of the dataset created by multiple imputation [Additional file 2, Tables 4b and 4d]. Estimates of association were robust to the worst-case scenario simulating missing not at random, except for perceived access to recreational areas [Additional file 2, Tables 4c and 4d].

## Discussion

To our knowledge, this is the first study of pregnant women combining objective and perceived measures of access to recreational areas and objectively recorded PA data. We found poor agreement between objective and perceived access to recreational areas. MVPA dropped between early- and mid-pregnancy, followed by an increase postpartum. Western women performed approximately 60 MVPA min/week more than South Asian women during early and mid-pregnancy, which increased to approximately 180 min/week more postpartum. Further, contrasted with Middle Eastern women, Western women accumulated more MVPA min/week during mid-pregnancy (approx. 60 min) and post-partum (approx. 150 min). Women with good objective access to recreational areas accumulated on average an additional 63 MVPA min/week compared to women with limited access. Further, a positive association was evident between perceived access to recreational areas and MVPA. Both objective and perceived access to recreational areas were independently associated with MVPA. Time point, ethnicity and SEP did not modify the associations between objective or perceived access and MVPA. In the adjusted models for the association between MVPA and access to recreational areas, less than 2 % of the variation in MVPA was attributed to differences among neighbourhoods.

### Strengths and weaknesses

The population-based Stork-G cohort is unique in Europe due to the large sample size, high-quality data, including objectively recorded measures of PA, and representativeness of the largest ethnic groups. Successful inclusion of ethnic minority women from sub-groups often excluded due to language difficulties enhances the external validity of the results. The combination of objectively recorded MVPA data and objective and perceived access to recreational areas enhances internal validity. The restriction of MVPA to bouts ≥10 min is likely more specific with respect to recreational PA compared with total accumulation of MVPA [33]. Adjustments for individual-level data on ethnicity, SEP, age and parity reduce the risk of spurious estimates of the association between neighbourhood-level objective access to recreational areas and MVPA [15].

However, the study has limitations. We did not collect data on the location of MVPA or participants’ decision to reside in their current neighbourhood. Neighbourhoods were defined according to postal codes, which may be too large geographically to represent neighbourhoods. The questionnaire included only selected items on perceptions of neighbourhood due to time constraints. Therefore, the component score used as a measure of perceived access to recreational areas did not originate from a validated scale. There was extensive missing data on MVPA (only 43 % of the study sample had MVPA data from postpartum). To avoid even larger reductions in sample size, we used MVPA data with ≥2 valid SWA days although 3–5 days of monitoring are recommended [34]. This was supported by sensitivity tests with ≥3 valid SWA days that yielded similar results. While it is impossible to determine if the mechanism underlying missing MVPA is missing at random or missing not at random, the inclusion of variables that predicted missing data on MVPA and MVPA values in the original models 1–3 supports the plausibility of the missing at random assumption [35]. These variables accompanied BMI as auxiliary variables in the multiple imputation procedure used to assess potential bias in the complete case analysis [35]. The agreement between the complete case analyses and the sensitivity analyses detailed in Additional file 2 supports the missing at random assumption and the results of the presented models, despite a high proportion of missing postpartum MVPA data.

### Association between objective and perceived access to recreational areas

The poor agreement between objective and perceived access to recreational areas concurs with previous studies [21]. Possible explanations may be that objective access was conditional on strict criteria for proximity and barriers or that it was measured at the neighbourhood-level while perceived access was measured at the individual-level. Objective access captures both pedestrian safety (i.e., access along an eligible walking route) and proximity to recreational areas. In contrast, perceived access reflects more nuanced participant assessments of the quality and appropriateness of recreational areas. As such, the two may complement each other and provide insight into perceived qualitative aspects of availability and access that are not readily captured by objective measures. The poor agreement may partly reflect that perceived properties of the environment, or affordances, are learnt through development and interactions with the physical environment [36] and that perceptions of the social and physical environment are entwined [37]. As a consequence, individuals with low self-efficacy for PA may perceive the same area as less accessible than individuals with high self-efficacy [15]. Alternatively, residents in disadvantaged neighbourhoods downgrade their aspirations [38] and consequently over-rate access to recreational areas.

### Overall development of MVPA

We are unaware of comparable longitudinal population-based studies of objectively recorded PA from pregnancy to postpartum. However, the observed drop in MVPA between early and mid-pregnancy concurs with studies based on self-reported PA [39, 40]. The observed increase in MVPA postpartum lends support to some studies based on self-reported PA [39, 40] but contradicts a recent systematic review that was primarily based on studies using self-reported PA, where the main finding was that PA did not increase after birth [41]. The observed increase in MVPA postpartum could potentially result from a higher proportion of missing data among participants who were less physically active.

### Ethnic differences in MVPA

The ethnic differences in MVPA concur with findings that non-Hispanic black women perform less MVPA during pregnancy than non-Hispanic white women [6]. In our study, Western women performed approximately one hour/week of MVPA more than South Asian women at both time points during pregnancy. The ethnic differences increased at three months postpartum, when Western women performed 182 MVPA min/week more than South Asian women and 154 MVPA min/week more than Middle Eastern women. To the best of our knowledge, it has not been previously reported that ethnic differences in MVPA persist during pregnancy into postpartum. At postpartum, the median (IQR) MVPA for Western women was 28.5 min/day. In the US-based Pregnancy Infection and Nutrition Postpartum Study, objective measures of MVPA indicated median MVPA three months postpartum was 13 min/day [42]. This discrepancy may reflect that women in Norway are entitled to a period of subsidized maternity leave after birth, which may provide more time for recreational activities compared to women in the US. The modest increase in MVPA between mid-pregnancy and postpartum among women with ethnic minority background is of particular concern. PA may reduce postpartum weight retention [43, 44] and subsequently reduce the risk of entering the next pregnancy as overweight/obese [45].

In our analysis, we did not explore possible mechanisms behind ethnic differences in MVPA. Other studies have indicated that SEP and acculturation (time since immigration) mediate ethnic disparities in leisure-time PA [46, 47]. PA behaviour develops in cultural contexts, and the ethnic differences in MVPA may reflect that recreational PA is less common or less accepted among Middle Eastern and South Asian women. Ethnic differences in MVPA may also be mediated by SEP. For example, ethnic minority women in the US perceived that they could not financially afford to free themselves from household chores, in contrast with their perception of Western women’s capacity [48]. We did not adjust for SEP in the present analyses, as this would misrepresent ethnic differences since SEP is, arguably, on the causal pathway between ethnicity and MVPA [49]. Future studies should explore mediators of ethnic differences in MVPA, such as SEP, discrimination, language barriers, migration history, acculturation and cultural preferences [49, 50].

### Associations between access to recreational areas and MVPA

On a weekly basis, participants with good access to recreational areas could accumulate up to 63 MVPA minutes more than participants with limited access, which represents 42 % of the recommended 150 MVPA min/week. Systematic reviews indicate a possible association between PA and access to recreational areas in non-pregnant populations [13, 14]. In the only study of which we are aware on associations between objective measures of park access and PA in pregnancy, no relationship was observed [20]. This discrepancy may result from the use of self-reported PA including types of PA that could not be carried out in the neighbourhood. In contrast, we employed an objective measure of MVPA, which is considered more valid than self-reported PA [7].

Securing good access to recreational areas is an important population-level strategy to promote health [51]. Our study indicates that the association between MVPA and objective good access to recreational areas, defined by an eligible walking route, extends to pregnant women. Given the importance of pregnancy for maternal and offspring health, securing good access to recreational areas that stimulate health-enhancing PA is of particular relevance. It has been hypothesized that PA is an important mediator of the association between access to recreational area and maternal physical and mental health during pregnancy [52]. Evidence of the effect of PA during pregnancy on preventing gestational diabetes, excessive gestational weight gain and maternal depressive symptoms underlines the importance of public health strategies that reach large groups of pregnant women [1, 53]. The rationale for securing good access to recreational areas during pregnancy is further supported by altered PA behaviour in pregnancy; participation in typical pre-pregnancy activities of at least moderate intensity declines during pregnancy while pregnant women accumulate a larger share of MVPA by brisk walking [12]. The persistent positive association, observed in the present study, between good objective access and MVPA throughout pregnancy and postpartum is thus encouraging; it indicates that access to recreational areas is important even in mid-pregnancy, when PA levels reach the lowest point.

The objective measure of good access to recreational areas was conditional on an eligible walking route with no barriers in terms of crossing roads/tracks. This may have captured that many women consider safety for themselves and their offspring as a prerequisite for being physically active during pregnancy and postpartum. A wide range of barriers to PA in pregnancy and postpartum are perceived to be outside women’s control, and strategies to integrate active living in daily routines may be effective [54]. Barriers such as childcare obligations and lack of time and energy are frequently reported [39], but accessible recreational areas may lessen PA barriers by facilitating active living in everyday life (e.g., walking with strollers). In line with the present study, previous studies outside pregnancy indicate that associations between accessibility and walking behaviour is not modified by ethnicity or SEP [55, 56]. In other words, initiatives to improve access to recreational areas may positively enhance health related behaviour, irrespective of ethnic and socio-economic background.

After adjustment for objective access, women with perceptions of high access to recreational areas accumulated on average an additional 31.5 min/week of PA compared to women with perceptions of low access. This translates to 21 % of the recommended weekly duration of MVPA. This independent association may manifest because women who regularly walk in their neighbourhood are more aware of typical recreational areas and hence perceive access to recreational areas differently than women less acquainted with their neighbourhood environment. Accordingly, by tapping into perceived qualitative dimensions of availability and access, we can gain insight into aspects not readily captured by objective measures.

The independent association between perception of recreational areas and MVPA support the potential impact of initiatives from staff at child health clinics to change pregnant women’s perceptions of their neighbourhood. For example, initiation of walking groups for women residing in the same neighbourhood could build social capital and simultaneously lower the threshold for exploring neighbourhood areas [57]. By getting to know neighbours and the physical environment, perceptions of access to recreational areas may become more positive. Pregnancy represents an important window of opportunity to support women who are contemplating a lifestyle change towards being more physically active. Policies that successfully provide and protect recreational areas in neighbourhoods that pregnant women perceive as safe and readily available may thus contribute to healthier pregnancies and long-lasting changes in PA behaviour.

### MVPA variation

Only a small proportion of the total MVPA variation could be attributed to differences among neighbourhoods. However, access to recreational areas could explain a large proportion of this variation across neighbourhoods, as indicated by the lower neighbourhood-level ICC in the model where objective access to recreational areas was defined as an explanatory variable, in contrast to the empty model [58]. The low neighbourhood-level ICC is likely a consequence of including relatively homogeneous neighbourhoods from three city districts in contrast to a random sample of neighbourhoods across Oslo.

## Conclusion

There is substantial evidence that physical attributes of neighbourhoods are positively associated with PA. The present findings contribute new information about the persistent positive association between access to recreational areas and MVPA during pregnancy and postpartum in multi-ethnic neighbourhoods. This is of particular public health relevance since a decline in MVPA occurs during this period, and factors that attenuate the magnitude of the decline may indirectly protect against adverse pregnancy and maternal health outcomes. Alarmingly, women with ethnic minority background accumulated substantially less MVPA compared to Western women at all time points. Thus, the present finding of a positive association between access to recreational areas and MVPA, irrespective of ethnicity and SEP, is encouraging and provides further support for population-based strategies to improve access to recreational areas. The causal mechanisms underlying the association between access to recreational areas and MVPA are not definitive. The observed poor agreement between objective and perceived access to recreational areas provides support for studies to identify factors that influence pregnant women’s perceptions of recreational areas, and to establish if approaches to influence their perceptions affect MVPA.

## Abbreviations

95 % CI, 95 % confidence interval; GIS, geographic information systems; GW, gestational week; ICC, intra-class correlation coefficient; IQR, interquartile range; MVPA, moderate-to-vigorous intensity physical activity; PA, physical activity; SD, standard deviation; SEP, socio-economic position; Stork-G, The Stork Groruddalen Cohort Study; SWA, SenseWear™ Pro3 Armband

## Additional files

Additional file 1: Drop-out flowchart. (PDF 382 kb) Additional file 2: Analyses of missing data and sensitivity. (PDF 328 kb) Additional file 3: Median (25th-75^th^ percentile) MVPA across visits. (PDF 191 kb) Additional file 4: Associations between access to neighbourhood recreational areas and moderate-to-vigorous intensity physical activity (minutes/day) including all co-variates. (PDF 233 kb)
