# Supplementary material for: Objectively recorded physical activity in pregnancy and postpartum in a multi-ethnic cohort: association with access to recreational areas in the neighbourhood
Source: Int J Behav Nutr Phys Act. 2016 Jul 7;13:78. doi: 10.1186/s12966-016-0401-y (PMC4936091; doi:10.1186/s12966-016-0401-y)
Supplement: Additional file 1: — Drop-out flowchart. (PDF 382 kb) [file 12966_2016_401_MOESM1_ESM.pdf]

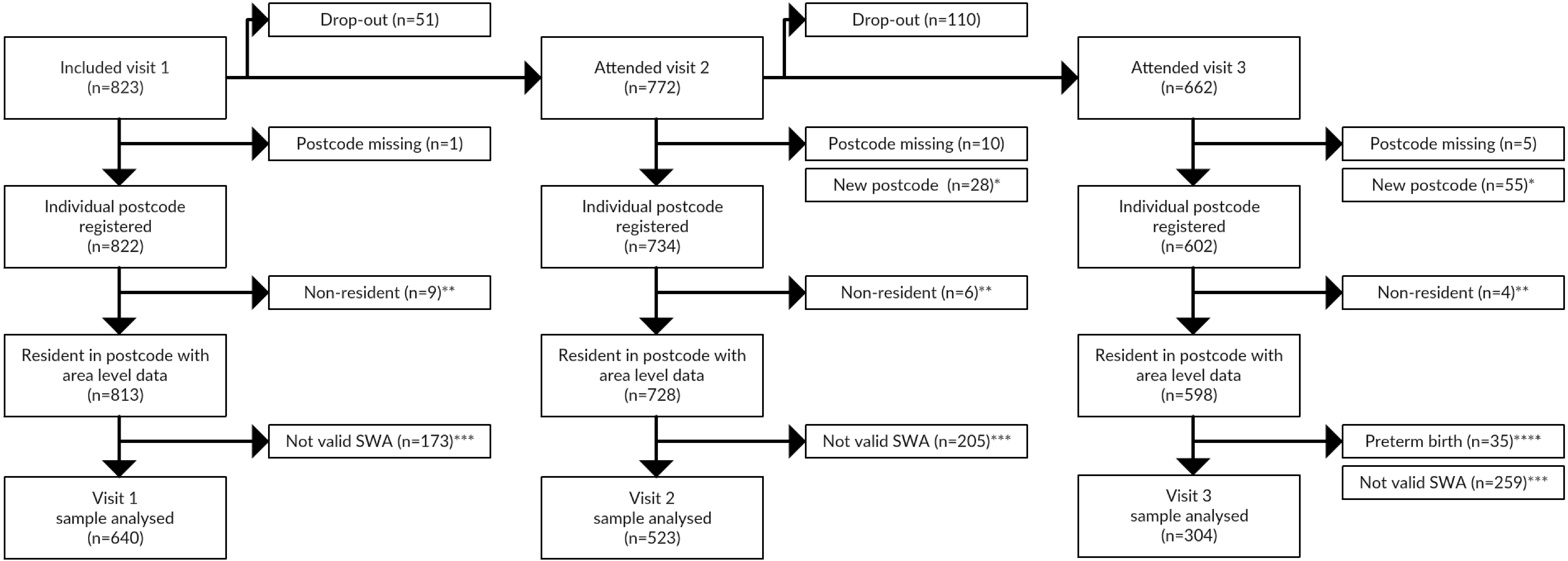

*Excluded if postcode not identical with postcode at visit 1
**Excluded if not resident within a postcode with available area level data
***Excluded if <2 days of at least 19.2 hr SWA weartime at visit
****Preterm birth if <gestational week 37
