# Supplementary material for: Objectively recorded physical activity in pregnancy and postpartum in a multi-ethnic cohort: association with access to recreational areas in the neighbourhood
Source: Int J Behav Nutr Phys Act. 2016 Jul 7;13:78. doi: 10.1186/s12966-016-0401-y (PMC4936091; doi:10.1186/s12966-016-0401-y)
Supplement: Additional file 2: — Analyses of missing data and sensitivity. (PDF 328 kb) [file 12966_2016_401_MOESM2_ESM.pdf]

Additional file 2: Analyses of missing data and sensitivity

Introduction

The originally fitted models presented in the paper built on analyses of data from three time points, restricted to participants with complete observations at each time point (complete case analysis).

The data material suffers from extensive missing data on MVPA. Missing data mechanisms describe the possible associations between measured variables and the probability of missing data [1]. Three missing mechanisms have been described [2]: **Missing completely at random (MCAR)** indicates that the probability of missing data on a variable Y is unrelated to other measured variables and the values of Y itself. **Missing at random (MAR)** indicates that the probability of missing data on a variable Y is related to one or several measured variable(s) in the model, but not the values of Y. **Missing not at random (MNAR)** is indicated if the probability of missing data on a variable Y is related to the values of Y after adjustments for other variables.

It is not possible to verify the missing data mechanism [1, 3], but it is recommended to explore whether the MAR assumption is plausible or not. Multiple imputation can, potentially, reduce bias in epidemiological models of association, but factors associated with both missing data and the outcome variable must be included as auxiliary variables in the imputation model [2].

It is recommended, also, to assess the robustness of inferences in analysis based on the MAR assumption to possible departures from the MAR assumption by assessing the models' sensitivity to MNAR mechanisms [4]. Numerous MNAR scenarios are possible and it is not feasible to assess sensitivity to all kinds of scenarios [5].

In this supplement, we describe analyses of missing data, and two forms of sensitivity analyses: First, we assessed bias arising from the complete case analysis by performing analyses of datasets generated by multiple imputation. Second, we assessed the models sensitivity to a specified MNAR mechanism: We tested the sensitivity to the worst-case scenario were we assumed that all participants with missing data on MVPA would have recorded 0 MVPA min/day. The analyses presented are based on recommendations by Sterne and colleagues [4].

1. Analyses of missing data and reasons for missing

Frequency of missing data

Data collected at three time points from 709 participants were included in the analysis. Hence, there was potential for 2127 data observations. Among the variables included in the original analyses, we observed extensive missing data on objectively recorded moderate-to-vigorous intensity physical activity (MVPA), which is the focus in this additional file. A flowchart [additional file 1] presents the dropout of participants from the Stork-Groruddalen study, and reported reasons for missing MVPA data at the each time point.

Table 1a presents the frequency and percentage of missing data, both with reference to the total number of observations and the total number of participants analysed.

Table 1a: Missing data presented as frequency and percentages of total observations (n=2127) and total participants (n=709)

	Observations with missing data (% of 2127 observations)	Participants with missing data (% of 709 participants)
Objectively recorded MVPA[1]	660 (31.0)	-
Objectively recorded MVPA time point 1	-	69 (9.7)
Objectively recorded MVPA time point 2	-	186 (26.2)
Objectively recorded MVPA time point 3	-	405 (57.1)
Gest./ postpartum week of PA[2] recording	142 (6.7)	-
Gestational week time point 2	-	30 (4.2)
Postpartum week time point 3	-	112 (15.8)
Season	142 (6.7)	-
Season time point 2	-	31 (4.4)
Season time point 3	-	111 (15.7)
Perceived access to recreational areas	-	36 (5.1)

[1]MVPA=Moderate-to-vigorous intensity physical activity
[2]PA=Physical activity

Missing pattern

With respect to MVPA the missing patterns show that missing data at time point 3 and missing data at time point 2 and 3 together represent 51 percent of the patterns. In total 34% of the sample had no missing data on MVPA (Table 1b).

Table 1b: Missing pattern of moderate-to-vigorous physical activity (n=709)

Frequency of pattern	Percent	Cumulative percent	Pattern[1] by time point 1	2	3
243	34.3	34.3	O	O	O
217	30.6	64.9	O	O	M
143	20.2	85.1	O	M	M
45	6.4	91.4	M	O	M
37	5.2	96.6	O	M	O
18	2.5	99.2	M	O	O
6	0.9	100.0	M	M	O

[1] O=observed; M=missing

Predictors of missing data and values on variables with missing data

We performed multiple logistic regression analyses to identify predictors of missing MVPA values at each time point, and predictors of incomplete MVPA data (i.e. missing on at least one time point). We explored predictors of missing data on perceived access to recreational areas by the same method. Parity status, socioeconomic position, and ethnicity were independently associated with missing (Table 1c).

Table 1c: Predictors of missing data

	Missing				
	MVPA				Perceived access to recreational areas
	Time point 1	Time point 2	Time point 3	Incomplete MVPA	
Parity	x[1]	x	x	x	
Socioeconomic position			x	x	x
Ethnicity			x	x	x
Body mass index					
Age					
Objective access to recreational areas					

[1] x indicates significant ($p<0.05$) association with missing data assessed by multiple logistic regression analysis

Association with MVPA min/day was determined by multiple linear regression analyses. Ethnicity and body mass index (BMI) were associated with MVPA min/day (Table 1d). Association with perceived access to recreational areas was determined by multiple logistic regression analyses, but no significant predictors were identified (Table 1d).

Table 1d: Predictors of observed values on variables with missing data

	Observed values			
	MVPA min/day			Perceived access to recreational areas (high/low)
	Time point 1	Time point 2	Time point 3	
Parity				
Socioeconomic position				
Ethnicity	x[1]	x	x	
Body mass index	x	x	x	
Age				
Objective access to recreational areas				

[1] x indicates significant ($p<0.05$) association with observed value

Except for BMI, all the predictors of missing data and observed values on variables with missing data were already included as confounders in the original models in the paper.

2. Comparison of participants by missing data on MVPA

Table 2a: Comparison of participants by missing MVPA at time point 3

	Valid MVPA[1] data from time point 3 (n=304)		Missing MVPA data from time point 3 (n=405)		
	Mean	*SD*	*Mean*	*SD*	P-value[2]
**Age at inclusion (years)**	30.7	4.6	29.6	5.0	<0.01
**BMI pre-pregnancy**	24.7	4.4	24.2	4.8	ns
	N	*%*	*N*	*%*	P-value[3]
**Objective access to recreational areas**					ns
Limited access	30	10	40	10	
Good access	274	90	365	90	
**Perceived access to recreational areas**					ns
Low perception	98	34	125	33	
High perception	195	66	256	67	
**Ethnicity**					<0.01
Western	158	52	150	37	
South Asian	58	19	110	27	
Middle Eastern	37	12	68	17	
Other ethnicity	51	17	77	19	
**Education**					<0.01
<10 years	37	12	71	18	
10-12 years	99	33	175	43	
University or college	166	55	158	39	
Unknown	2		1		
**Occupation**					0.02
Elementary occup./homemakers	71	24	122	31	
Clerical/care occupations	103	34	142	36	
Manager/degree occupations	128	42	129	33	
Unknown	2		12		
**Parity**					ns
None (nulliparous)	127	42	204	50	
1 (uniparous)	116	38	130	32	
≥2 (multiparous)	61	20	71	18	
**Housing**					ns
Flat	235	79	331	83	
Semi-detached or detached housing	64	21	69	17	
Unknown	5		5		
**Health pre-pregnancy**					ns
Poor/not too good	27	9	44	11	
Good	154	51	200	50	
Very good	120	40	158	39	
Unknown	3		3		
**Smoking behaviour pre-pregnancy**					ns
Non-smoker	249	82	330	82	
Daily or irregular smoker	54	18	72	18	
Unknown	1				

[1] Moderate-to-vigorous intensity physical activity data based on ≥2 valid days
[2] Independent samples t-test
[3] Chi-square test

Selective dropout at time point 3 was evident. In addition to non-Western women and women with ≤12 year's education, we observed that homemakers and women with elementary occupations had a higher dropout rate than women in other occupations (Table 2a).

3. Sensitivity analysis methods

Since predictors of missing MVPA and MVPA values were included in the original models as confounders, the plausibility of the MAR assumption was supported. However, since BMI predicted MVPA (Table 1d) but was not considered to be a relevant confounder in the original models, inclusion of BMI as an auxiliary variable in the multiple imputation analysis was warranted. Inclusion of BMI would strengthen the plausibility of the MAR assumption of the models based on imputed data. The procedure is described in this section, and results are presented in section 4.

Multiple imputation

As STATA 13 does not account for clustering of data in the imputation phase, we used the software REALCOM-IMPUTE (http://www.bristol.ac.uk/cmm/software/realcom/imputation.html), which accounts for 2-level data structures. Since the software does not allow for more than two levels, the imputation phase did not account for the clustering of participants within neighbourhoods. Effect estimates from complete case analyses of models not taking into account neighbourhood-level clustering matched the effect estimates from the original three-level models presented in the paper. Hence, we considered it appropriate to use 2-level models in the sensitivity analyses.

The variables included in the imputation model were imported to REALCOM-IMPUTE. Following imputation, the imputed datasets were imported into STATA 13, with which we pooled the estimates from the imputed datasets and performed subsequent analyses.

For the imputation, we defined repeated measurements (i.e. time point 1-3) as level 1, while participants were defined as level 2. To reduce sampling variability we imputed 50 datasets [4]. Five hundred burn-in iterations preceded the first imputed dataset to allow sufficient time for the parameter estimates to stabilize [1], and 500 between-imputation iterations separated the remaining 49 imputed datasets (in total 25,000 iterations) [6, 7].

Variables with missing data were defined as response variables in the imputation model (MVPA, week, season and perceived access to recreational areas) [8]. Variables with complete data were included as auxiliary variables (socioeconomic position, age, time point, ethnicity, parity, objectively recorded access to recreational areas and BMI) [4]. A random intercept term for each individual was also included. Binary and categorical response variables were included in the imputation model in accordance with the prescribed procedure [8]. While MVPA was positively skewed, we preferred the original variable to a normalized transformation in the imputation model, since empirical evidence indicates that normality violations do not pose serious threats to the accuracy of multiple imputation parameter estimates [1]. We included no statistical interactions in the imputation model

Worst-case scenario

We performed worst-case sensitivity analyses to test the robustness of the estimates from the original model and the multiple imputation models to departures from the MAR assumption. The sensitivity analyses were performed by assessment of a specified worst-case scenario where we imputed missing values on MVPA by replacing the missing value with values with 0 MVPA

min/day. This worst-case scenario originated from the hypothesis that participants with missing MVPA data "would have" recorded no MVPA in bouts of at least 10 minutes.

With respect to missing data on perceived access to recreational areas, the same procedure was used, and missing values were replaced with the score 0, i.e. low perception of access to recreational areas. For missing data on season and week we kept the imputed values generated by multiple imputation, since these were considered to vary randomly.

4. Results of sensitivity analyses

We have presented estimates of the associations between explanatory variables and MVPA for the four original models (complete case analyses) in tables 4a to 4d, alongside estimates based on multiple imputation and the worst-case scenario.

Table 4a: Association between ethnicity and moderate-to-vigorous physical activity (min/day)

	Original model [1,3]		Multiple imputation model [2,3]		Worst-case scenario model [3,4]	
	β	95% CI	β	95% CI	β	95% CI
**Ethnicity (ref: Western)**						
South Asian	-9.17**	-15.19 ,-3.15	-9.32**	-15.30 ,-3.34	-7.63**	-12.86 ,-2.41
Middle Eastern	-6.05	-13.11 ,1.00	-7.20*	-14.39 ,-0.01	-6.11*	-12.22 ,-0.01
Other Ethnicity	-7.58*	-14.17 ,-0.99	-8.23*	-14.78 ,-1.68	-7.88**	-13.59 ,-2.17
**Time point (ref: Early pregnancy)**						
Mid-pregnancy	-8.64**	-12.75 ,-4.54	-8.15**	-12.48 ,3.83	-10.65**	-14.23 ,-7.07
Postpartum	14.82**	10.11 ,19.54	12.46**	7.37 ,17.53	-3.17	-6.75 ,0.40
**Interactions (Ref: Western * Early pregnancy)**						
South Asian * Mid-pregnancy	1.12	-5.97 ,8.20	0.88	-6.54 ,8.31	1.65	-4.37 ,7.68
South Asian * Postpartum	-16.67**	-25.53 ,-7.81	-8.10	-16.65 ,0.44	-8.90**	-14.91 ,-2.89
Middle Eastern * Mid-pregnancy	-2.26	-10.78 ,6.25	-1.20	-9.81 ,7.43	-0.96	-8.03 ,6.12
Middle Eastern * Postpartum	-16.14**	-26.68 ,-5.61	-8.73	-18.87 ,1.42	-9.14*	-16.22 ,-2.05
Other * Mid-pregnancy	2.73	-5.10 ,10.57	2.07	-5.93 ,10.07	4.39	-2.21 ,11.01
Other * Postpartum	-14.03**	-23.51 ,-4.55	-7.58	-16.81 ,1.65	-6.46	-13.06 ,0.14

[1] Three-level linear mixed effects regression models
[2] Two-level linear mixed effects regression model based on 50 datasets created by multiple imputations
[3] Adjusted for gestational/postpartum week, socioeconomic position, ethnicity, season, parity, age
[4] Worst-case scenario: For participants with missing MVPA data the imputed value is 0
* $p<0.05$
** $p<0.01$

With respect to ethnic differences, the multiple imputation model supported the original model, replicating differences in MVPA min/day between Western women and South Asian women and women with other ethnicity, respectively (Table 4a). Although the interaction between ethnicity and time point was not significant in the multiple imputation model, it was close to the result of original model, as the confidence intervals for the interaction between ethnicity and postpartum indicated a trend towards a widening gap postpartum between Western women and women from the other ethnic groups.

The worst-case scenario model indicated that the ethnic differences in early pregnancy were robust to the potential MNAR mechanism (Table 4a). Significant interactions between time point

and ethnicity was demonstrated with respect to South Asian and Middle Eastern women, but the effect size was smaller compared with the original model.

Table 4b: Association between objective access to recreational areas and moderate-to-vigorous physical activity (min/day)

	Original model 1 [1,3]		Multiple imputation model 1 [2,3]		Worst-case scenario model 1 [3,4]	
	β	95% CI	β	95% CI	β	95% CI
**Objective access to recreational areas (ref: limited access)**						
Good access	9.14**	2.66 ,15.62	9.17**	3.13, 15.23	6.59*	1.60 ,11.58

[1] Three-level linear mixed effects regression models
[2] Two-level linear mixed effects regression model based on 50 datasets created by multiple imputations
[3] Adjusted for gestational/postpartum week, socioeconomic position, ethnicity, season, parity, age
[4] Worst-case scenario: For participants with missing MVPA data the imputed value is 0.
* p<0.05
** p<0.01

With respect to the association between objective access to recreational areas and MVPA, there were no substantial differences between the results in the original model and the multiple imputation model (Table 4b and 4d). The worst-case scenario model showed that the association between objective access to recreational areas and MVPA remained significant and within the confidence intervals of the estimates in the original model and the multiple imputation model. The worst-case scenario model supported the robustness of the association between objective access to recreational areas and MVPA.

Table 4c: Association between perceived access to recreational areas and moderate-to-vigorous physical activity (min/day)

	Original model 2 [1,3]		Multiple imputation model 2 [2,3]		Worst-case scenario model 2 [3,4]	
	β	95% CI	β	95% CI	β	95% CI
**Perceived access to recreational areas (ref: Low perception)**						
High perception	4.75*	0.68 ,8.82	4.43*	0.57, 8.28	2.67	-0.44 ,5.78

[1] Three-level linear mixed effects regression models
[2] Two-level linear mixed effects regression model based on 50 datasets created by multiple imputations
[3] Adjusted for gestational/postpartum week, socioeconomic position, ethnicity, season, parity, age
[4] Worst-case scenario: For participants with missing MVPA data the imputed value is 0. For participants with missing perceived access to recreational area the imputed value is set to low perception
* p<0.05
** p<0.01

The association between perceived access to recreational areas and MVPA remained significant in the multiple imputation model, in line with the original model (Tables 4c and 4d). The worst-case

scenario model indicated that the associations observed in the original model and the multiple imputation model were not robust to potential departures from the MAR assumption; the β-coefficients supported the positive trend, but the associations were not statistically significant in the worst-case scenario model.

Table 4d: Mutually adjusted perceived and objective access to recreational areas and association with moderate-to-vigorous physical activity (min/day)

	Original model 3 [1,3]		Multiple imputation model 3 [2,3]		Worst-case scenario model 3 [3,4]	
	β	95% CI	β	95% CI	β	95% CI
**Objective access to recreational areas (ref: limited access)**						
Good access	9.06**	2.39 ,15.74	8.77**	2.71, 14.83	6.40*	1.41 ,11.40
**Perceived access to recreational areas (ref: Low perception)**						
High perception	4.40*	0.34 ,8.45	4.07*	0.23, 7.92	2.49	-0.61 ,5.59

[1] Three-level linear mixed effects regression models
[2] Two-level linear mixed effects regression model based on 50 datasets created by multiple imputations
[3] Adjusted for gestational/postpartum week, socioeconomic position, ethnicity, season, parity, age
[4] Worst-case scenario: For participants with missing MVPA data the imputed value is 0. For participants with missing perceived access to recreational area the imputed value is set to low perception
* $p<0.05$
** $p<0.01$

5. Conclusion

Plausibility of the missing at random assumption (multiple imputation)

The observation that missing data and observed values on variables with missing data were systematically associated with other measured variables show that there is no support for the MCAR assumption (Table 1c and 1d). In accordance with previous studies, we observed that several factors (i.e. socioeconomic position, ethnicity and age) predicted missing data [9]. The inclusion of predictors of missing data and values on MVPA in the original models and in the multiple imputation models supported the plausibility of the MAR assumption of these models. Probably, the addition of BMI as an auxiliary variable in the multiple imputation procedure further strengthened the plausibility of the MAR assumption of the multiple imputation models. While interactions between ethnicity and time point were not significant in the multiple imputation model (Table 4a), the overall picture shows that the original models and the multiple imputation models yielded similar results, suggesting that missing data did not bias the results of the original models.

Robustness to potential missing not at random (worst-case scenario)

The original model of the association between ethnicity and MVPA at time point 1 was robust to the specified MNAR mechanism (Table 4a). The effect estimate given the worst-case scenario remained significant and within the 95% confidence interval observed in the original model. The interaction between ethnicity (South Asian and Middle Eastern women) and time point observed

in the original model was also replicated, indicating that the interaction effect was robust to the specified MNAR mechanism.

The association between objective access to recreational areas and MVPA was robust to the specified MNAR mechanism (Table 4b and 4d). However, the effect estimates were attenuated in model 1 and 3, but in both tests, they remained within the 95% confidence intervals observed in the original models and the imputation models. The association between perceived access to recreational areas and MVPA showed a positive trend, but the association was no longer significant (Table 4c and 4d), indicating that the original model estimate of this association is less robust to the specified MNAR mechanism.

References

1. Enders, C.K., *Applied missing data analysis*. Methodology in the social sciences. 2010, New York: Guilford Press.
2. Spratt, M., et al., *Strategies for multiple imputation in longitudinal studies*. American journal of epidemiology, 2010. **172**(4): p. 478-487.
3. Molenberghs, G., et al., *Missing data*, in *Handbook of Epidemiology*, W. Ahrens and I. Pigeot, Editors. 2014, Springer New York: New York, NY. p. 1283-1335.
4. Sterne, J.A., et al., *Multiple imputation for missing data in epidemiological and clinical research: potential and pitfalls*. BMJ, 2009. **338**: p. b2393.
5. Groenwold, R.H., et al., *Dealing with missing outcome data in randomized trials and observational studies*. American journal of epidemiology, 2012. **175**(3): p. 210-217.
6. Enders, C.K., *Analyzing longitudinal data with missing values*. Rehabil Psychol, 2011. **56**(4): p. 267-88.
7. Carpenter, J.R., H. Goldstein, and M.G. Kenward, *REALCOM-IMPUTE software for multilevel multiple imputation with mixed response types*. Journal of Statistical Software, 2011. **45**(5): p. 1-14.
8. Bartlett, J. *Using REALCOM Impute and Stata*. 2011.
9. Razzaghi, H., et al., *Impact of Missing Data for Body Mass Index in an Epidemiologic Study*. Matern Child Health J, 2016.
