# Supplementary material for: Objectively recorded physical activity in pregnancy and postpartum in a multi-ethnic cohort: association with access to recreational areas in the neighbourhood
Source: Int J Behav Nutr Phys Act. 2016 Jul 7;13:78. doi: 10.1186/s12966-016-0401-y (PMC4936091; doi:10.1186/s12966-016-0401-y)
Supplement: Additional file 3: — Median (25th-75th percentile) MVPA across visits. (PDF 191 kb) [file 12966_2016_401_MOESM3_ESM.pdf]

Additional file 3: Median MVPA minutes/day with 25th-75th percentile at time points 1-3

	Time-point 1 (early pregnancy)		Time-point 2 (mid-pregnancy)		Time-point 3 (post-partum)	
	Median	P25-P75	Median	P25-P75	Median	P25-P75
**Objective access**						
Limited	8.0	(2.5 - 26.0)	4.0	(0.0 - 14.7)	12.5	(3.7 - 23.3)
Good	14.5	(4.3 - 33.3)	9.2	(2.5 - 22.0)	19.5	(5.8 - 48.0)
**Perceived access**						
Low perception	9.5	(3.3 - 25.5)	7.4	(0.0 - 17.0)	16.4	(4.0 - 40.8)
High perception	16.8	(5.3 - 35.0)	10.3	(2.8 - 24.5)	20.6	(7.0 - 48.5)
**Ethnicity**						
Western	20.3	(7.8 - 37.0)	11.5	(3.7 - 26.0)	28.5	(12.8 - 61.3)
South Asian	8.8	(2.8 - 19.0)	4.8	(0.0 - 17.0)	6.4	(0.0 - 23.0)
Middle Eastern	9.8	(2.8 - 25.3)	5.3	(0.0 - 13.7)	5.8	(0.0 - 21.5)
Other	10.4	(2.7 - 28.3)	8.3	(0.0 - 18.3)	12.0	(3.8 - 26.0)
**Completed education**						
University/collected	18.9	(6.3 - 36.5)	11.7	(3.3 - 25.3)	23.7	(8.5 - 48.4)
10-12 years education	10.5	(3.3 - 27.7)	5.5	(0.0 - 15.5)	18.0	(5.5 - 42.0)
<10 years education	8.3	(1.8 - 31.8)	6.8	(0.0 - 16.7)	5.0	(0.0 - 16.5)
**Occupation**						
Manager/degree occupations	21.6	(8.4 - 40.6)	13.4	(4.5 - 26.2)	26.3	(11.3 - 54.7)
Clerical/care occupations	10.8	(3.3 - 25.3)	7.5	(0.0 - 17.0)	17.7	(6.0 - 37.7)
Elementary occupations or homemakers	9.2	(2.5 - 25.8)	4.0	(0.0 - 15.4)	6.8	(0.0 - 27.7)
**Parity**						
Nullipara	15.6	(5.0 - 36.3)	11.0	(1.8 - 24.8)	23.0	(7.2 - 56.7)
Uni/multipara	12.7	(3.3 - 27.7)	8.0	(2.0 - 17.5)	15.5	(4.0 - 37.0)
**Season**						
Winter	12.5	(2.8 - 30.5)	6.5	(0.0 - 14.9)	15.3	(5.0 - 31.0)
Spring	14.5	(5.0 - 33.0)	9.2	(3.0 - 22.3)	20.3	(6.8 - 59.7)
Summer	14.2	(5.5 - 35.0)	12.0	(2.8 - 27.7)	17.7	(5.8 - 43.3)
Autumn	12.8	(3.7 - 31.1)	7.2	(0.0 - 18.3)	22.0	(4.6 - 46.5)
