# Supplementary material for: Objectively recorded physical activity in pregnancy and postpartum in a multi-ethnic cohort: association with access to recreational areas in the neighbourhood
Source: Int J Behav Nutr Phys Act. 2016 Jul 7;13:78. doi: 10.1186/s12966-016-0401-y (PMC4936091; doi:10.1186/s12966-016-0401-y)
Supplement: Additional file 4: — Associations between access to neighbourhood recreational areas and moderate-to-vigorous intensity physical activity (minutes/day) including all co-variates. (PDF 233 kb) [file 12966_2016_401_MOESM4_ESM.pdf]

Additional file 4: Associations between access to neighbourhood recreational areas and moderate-to-vigorous intensity physical activity (minutes/day) including all co-variates

	Model 1[a]		Model 2[a]		Model 3[a]	
	β	95% CI	β	95% CI	β	95% CI
Fixed effects						
**Objective access to recreational areas (ref: limited access)**						
Good access	9.14**	2.66 , 15.62			9.06**	2.39 , 15.74
**Perceived access to recreational areas (ref: Low perception)**						
High perception			4.75*	0.68 , 8.82	4.40 *	0.34 , 8.45
**Time point (ref: Early pregnancy)**						
Mid-pregnancy	-8.15**	-10.94 , -5.36	-8.28**	-11.16 , -5.40	-8.23**	-11.11 , -5.36
Post-partum	7.04**	3.65 , 10.43	7.49**	4.01 , 10.98	7.51**	4.03 , 11.00
**Gestational-/post-partum week (time-point mean-centred)**	0.09	-0.47 , 0.66	0.09	-0.50 , 0.68	0.10	-0.48 , 0.69
**Socio-economic position**	2.44*	0.18 , 4.70	2.85 *	0.48 , 5.21	2.89*	0.53 , 5.24
**Ethnicity (ref: Western)**						
South Asian	-9.72**	-14.88 , -4.56	-10.00**	-15.32 , -4.68	-10.07**	-15.34 , -4.79
Middle Eastern	-6.84**	-13.06 , -0.62	-5.26	-11.66 , 1.15	-5.43	-11.80 , 0.94
Other ethnicity	-6.99*	-12.54 , -1.44	-6.64*	-12.36 , -0.91	-6.32*	-12.02 , -0.62
**Season (ref: Winter)**						
Spring	6.08**	2.38 , 9.77	6.21**	2.40 , 10.03	6.07**	2.26 , 9.88
Summer	3.74	-0.11 , 7.59	3.80	-0.17 , 7.76	3.78	-0.19 , 7.74
Autumn	4.25*	0.59 , 7.91	4.67*	0.90 , 8.44	4.59*	0.83 , 8.36
**Parity (ref: Nulliparous)**						
Parous	-1.87	-6.03 , 2.30	-1.05	-5.27 , 3.18	-1.12	-5.33 , 3.08
**Age**	-0.53*	-0.97 , -0.09	-0.56*	-1.01 , -0.11	-0.54*	-0.99 , -0.09
Random effects						
**Intra-class correlation (%) neighbourhood level**	0.7		1.6		1.1	
**Intra-class correlation (%) individual level**	40.0		39.0		38.5	

[a] Three-level linear mixed effects regression models
* p<0.05
** p<0.01
